# The processes involved in the establishment of user-provider partnerships in severe psychiatric illnesses: a scoping review

**DOI:** 10.1186/s12888-022-04303-5

**Published:** 2022-10-26

**Authors:** Aletta Boerkoel, Mats Brommels

**Affiliations:** 1 Institute for Community Medicine, University Medicine Greifswald, Greifswald, Germany; 2 Department for Learning, Informatics, Management and Ethics, Karolinska Institutet, Solna, Sweden

**Keywords:** User-provider partnerships, Person-centred care, Psychiatry, Health services research, User-led care

## Abstract

**Purpose:**

With the rising relevance of person-centred care, initiatives towards user-led decision making and designing of care services have become more frequent. This designing of care services can be done in partnership, but it is unclear how. The aim of this scoping review was to identify for mental health services, what user-provider partnerships are, how they arise in practice and what can facilitate or hinder them.

**Methods:**

A scoping review was conducted to obtain a broad overview of user provider partnerships in severe mental illness. Data was inductively analysed using a conventional content analysis approach, in which meaning was found in the texts.

**Results:**

In total, 1559 titles were screened for the eligibility criteria and the resulting 22 papers found relevant were analysed using conventional content analysis. The identified papers had broad and differing concepts for user-provider partnerships. Papers considered shared decision making and user-involvement as partnerships. Mechanisms such as open communication, organisational top-down support and active participation supported partnerships, but professional identity, power imbalances and stress hindered them. Users can be impeded by their illness, but how to deal with these situations should be formalised through contracts.

**Conclusion:**

The field of research around user-provider partnerships is scattered and lacks consensus on terminology. A power imbalance between a user and a provider is characteristic of partnerships in mental healthcare, which hinders the necessary relationship building allowing partnerships to arise. This power imbalance seems to be closely linked to professional identity, which was found to be difficult to change.

**Supplementary Information:**

The online version contains supplementary material available at 10.1186/s12888-022-04303-5.

## Introduction

Designing healthcare with the patient as a partner, thereby enabling patients to use their expertise to collaboratively reach their health goals, could be considered the ultimate form of patient centred healthcare [1, 2]. The building of trust that is needed to achieve this, is where healthcare providers and researchers are currently exploring the possibilities and opportunities [3–5]. Intuitively, this is a logical further development of personalised medicine, patient centred healthcare and shared decision making — themes that more and more hospitals are advertising as key treatment components on their websites (https://www.karolinska.se/for-patienter/patientinflytande/). A person-centred approach to care means putting the person’s views at the centre of care with the goal to achieve a meaningful life, and can therefore not be achieved without the person being treated participating actively [6, 7].

Even though many healthcare providers advertise providing patient centred healthcare [8], few examples of this can be found in mental healthcare. Person centred mental healthcare, or mental healthcare in partnership, is not clearly defined, making it challenging to identify the relevant literature. Terminology like shared decision making, user involvement, user participation, and user partnerships are used interchangeably, creating a diverse field of research lacking consensus. Within this field, only Bee, et al. [9] and Gondek, et al. [10] have tried to identify barriers and facilitators for user-led care planning in mental healthcare, so that a greater understanding could be created on how it can be implemented. This, however, is not focussing on user-provider partnerships. Within the concept of person centred care, designing care together with the user, also proposed as “co-care” [2], ought to be considered separately, as it is considered the ultimate form of person centredness [1, 11]. Because of our lack of understanding of co-designed care in mental health, scoping the field is a useful way to gain more understanding. The current project therefore aims to further explore the field of user-provider partnerships, their forms, underlying processes, and factors promoting and hindering partnerships to evolve.

## Methods

To explore the field of user-provider partnerships a scoping review was performed. Within healthcare research a scoping review is an excellent methodology to rapidly identify key concepts within the field of interest [12–14]. The methodology was taken from Arksey and O'Malley [12] with more detailed specification on the methodology provided by Levac, et al. [13] and the PRISMA-SCR statement [15]  to ensure the rigour of the qualitative analysis. The design evolved during data collection using an iterative approach, such that when new relevant approaches or frameworks for analysis emerged from the data, they were considered and adopted where appropriate. Because of the iterative approach, no review registration was made.

### Stage 1: identifying the research question

The population of interest for this scoping review is people with long term mental health problems, as for them it is more important to learn to live with one’s symptoms and manage them, rather than aim for a cure. Some examples of user-led care in schizophrenia were known to the researchers, hence the decision to focus on severe mental illness was made [16, 17]. To explore what processes are involved in the establishment of patient-provider partnerships in the recovery of patients with severe psychiatric disorders, three research questions were chosen.

1. What classifications can be used for patient-provider partnerships in a mental health context?
2. What are the mechanisms that establish patient-provider partnerships?
3. What factors can be identified that hinder or facilitate patient-provider partnerships?

### Stage 2: identifying relevant studies

Web of Science, PubMed, and PsycInfo were searched using Boolean operators. No searches for grey literature were done. The two authors discussed the concept of user-provider partnerships in mental healthcare in order to reach a consensus view. The consensus view led to search strategies based on the following concepts: “psychiatric disorder”, “patient/provider- participation/involvement/partnership”, “shared decision making”, and “collaborative care” were drafted in collaboration with a librarian. Trial searches were conducted to verify the usefulness of the identified key terms and to identify any unfamiliar terms that had not been thought of yet. The complete search strategies can be found in the supplementary material.

### Stage 3: study selection

As familiarity with the field of research increased, inclusion and exclusion criteria were decided post-hoc (see Table 1). Triangulation across data identified titles contradicting the initial inclusion and exclusion criteria, such as Bee et al. [9] or Bradley [18]. Based on these findings the inclusion criteria were adapted. The final inclusion and exclusion criteria were evaluated by both authors in relation to the list of abstracts, to verify their utility and minimise selection bias.

**Table 1:** Inclusion and exclusion criteria used during abstract screening

***Inclusion criteria***	***Exclusion criteria***
Publication language: English	No abstracts available
End date: 21.02.2020, update until 14.09.2021	Books
Time period: 2015 onwards	Randomised controlled trials
Context: Participation of users with one or more of the following: schizophrenia, schizoaffective disorder, bipolar I, bipolar II, anorexia nervosa, bulimia nervosa, major depressive disorder [19, 20], ‘severe mental illness’, or ‘psychiatric care’	Research with children as the user
Study design: All types of studies were included apart from randomised controlled trials	Not focussing on partnerships
Papers discussing recovery	Evaluation of intervention or change in treatment processes unless discussing the implementation process
Papers mention partnership component with provider, i.e. shared decision making, co-production, collaboration, co-creation, partnership, co-design	Focus on medication as a treatment
Journal is specifically for psychiatry, but title does not mention illness	Family/carer partnerships with provider
	E-health as a self-support tool

Titles were screened twice for combinations of key terms by a single researcher: when a mental disorder was mentioned in combination with one of the partnership terms (“patient provider- participation -involvement -partnership”, “co-production, -design, -creation”, “shared decision making”, and “collaborative care”) the title was included for the abstract screening round. Titles were screened twice to ensure the quality of the screening, the results of these screening outcomes were then compared and combined to the final list of titles for abstract screening. The second step was to screen abstracts based on the inclusion criteria (see Table 1). If it was unclear during the abstract screening if a paper was of relevance, the paper was included for full text screening as outlined in Arksey and O'Malley [12] as abstracts do not always reflect the true meaning of a study. During full text screening, a paper was included when it focused on partnerships between users and providers, when there was a case of uncertainty in inclusion, the second author reviewed the paper and a joint decision was made on inclusion or exclusion.

### Stage 4: charting the data

Data was charted following a conventional content analysis approach using pen and paper. Conventional content analysis is a methodology that is data or text driven and creates meaning out of single units of information [21]. The conventional content analysis was chosen as a charting methodology because this project aimed to create an understanding of the field and it allows for a breadth of concepts to be identified. The texts were read thoroughly a first time to become familiar with the content, this is considered immersing oneself in the content of the papers, like one would with a literary novel [21]. During the second reading, words and sentences were highlighted that capture key thoughts or concepts in the text. For the third reading, the researcher’s thoughts, impressions, and initial analysis, concerning the previously highlighted words and sentences were noted down. As the reading and reflecting continued, codes were formed that encompassed more than one thought.

### Stage 5: collating, summarizing, and reporting results

The identified codes were combined into broader categories when they showed relationships. This was done by writing the codes onto individual pieces of paper and clustering codes with similar meanings. Finally, these broader categories were collated and abstracted into meaningful themes [22]. The data analysis was initially done by AB, and thereafter discussed in detail with MB to ensure the combinations identified were reflected upon in the context of the researcher’s background. As outlined in Arksey and O'Malley [12], the data was presented in a way that ensured that the purpose of the scoping review was clearly represented, and findings were connected with its implications for the field.

## Findings

**Fig. 1:**
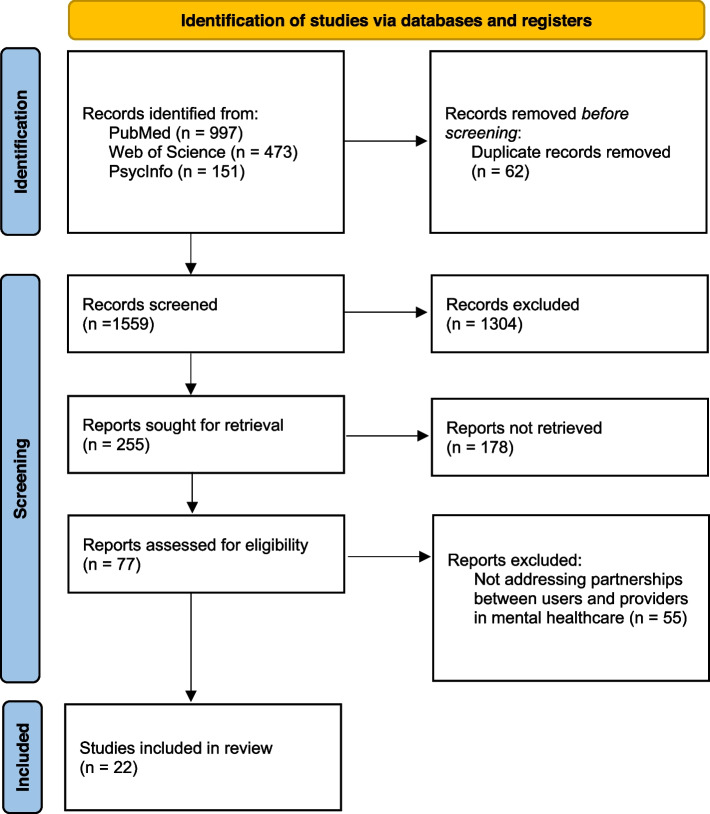
PRISMA flowchart of selection of titles. Flowchart adapted from Page et al. [23]

Data for screening was retrieved on 21.02.2020. This initial search of the database yielded 1192 titles. After duplicates were removed, a total of 1144 titles remained. An update on the search was retrieved on 09.09.2021, resulting in another 429 titles, out of which 14 duplicates were removed. A total of 1559 titles were screened. As shown in Fig. 1, the resulting 22 texts were analysed. The complete list of included articles and their corresponding information can be found in Table 2.

**Table 2:** Identified papers and their characteristics such as country of origin, methodology, target population and type of partnership

Author	Country	Methodology	Target population	Type of partnership	Setting
Hamann et al. (2016) [24]	Germany	Focus groups	Schizophrenia, bipolar disorder patients, and psychiatrists	Patient involvement, shared decision making	Acute psychiatric care, inpatient, outpatient—public and private care
Terry and Coffey. (2019) [25]	Wales	Interviews and focus groups	Mental health nurses, nursing students, and service users	Service user involvement	Mental health nursing care settings
Soderberg et al. (2019) [26]	Sweden	Interviews	Mental healthcare personnel	Patient participation	Forensic psychiatric care
Klausen, et al. (2017) [27]	Norway	Interviews	Service users	User involvement, shared decision making	Community mental healthcare
Dahlqvist et al. (2015) [28]	Sweden	Focus groups and interviews	Service users with schizophrenia, bipolar disorder, depression, and similar disorders	Service user participation, shared decision making	Mental health services in Sweden
Wallace et al. (2016) [29]	England	Focus groups and interviews	Service users with schizophrenia, bipolar disorder, depression, and similar disorders	Service user recovery promoting relationship/partnerships	Community mental health teams pro-recovery intervention
Schön et al. (2018) [30]	Sweden	Intervention, implementation, process evaluation	Mental health staff	User involvement, shared decision making	Psychiatric units in and outpatient
Ahmed et al. (2016) [31]	United States of America	Literature review	Schizophrenia patients	Care recipient involvement, partnership, engagement	Contemporary psychiatric services and recovery treatment for schizophrenia
Pelletier et al. (2015) [32]	Canada	Participatory action research	Schizophrenia patients	Patient partnership	Primary care
Sather et al. (2019) [33]	Norway	Focus group	Former severe mental illness inpatients	Patient participation, shared decision making	Transition from district psychiatric hospital centres to community mental health services
Miller et al. (2017) [34]	United States of America and Scotland	Literature review	Patients with severe mental illness	Person centred care planning	Person-centred care planning
Millar et al. (2016) [35]	Global/UK	Evolutionary concept analysis	Mental health care	Service user involvement	Service user involvement
Lwembe et al. (2017) [36]	England	Interviews and focus groups	Black and minority ethnic communities users with severe mental illness	Co-production, partnership	Community mental health service co-production intervention
Bentley et al. (2018) [37]	United States of America	Focus group and training protocol for shared decision making	Psychiatry residents	Shared decision making	Psychiatry resident training clinical setting
Ellegaard et al. (2018) [38]	Denmark	Interviews and focus groups	Professionals working with PCA program	Patient partnership	Public mental health hospitals—patient controlled admissions psychosis/affective disorders
Terp et al. (2016) [39]	Denmark	Co-design/ participatory design process reporting	Schizophrenia patients	Young adult user participation and engagement	Co-design to develop smartphone application
Kortteisto et al. (2018) [40]	Finland	Questionnaire	Mental health professionals (majority nurses)	Service user involvement	Psychiatric in and outpatient care in hospital districts
Brooks et al. (2019) [41]	England	Process evaluation through interviews	Mental health service users, professionals, and carers	Service user involvement	Shared Decision Making training package in secondary mental health care
Gunasekara et al. (2017) [42]	Australia	Interviews	Service users and psychiatrists	Service user involvement	Psychiatry treatment settings
Huang et al. (2020) [43]	China	Interviews	Schizophrenia patients	Shared decision making	Psychiatry department of a tertiary hospital
Larsen et al. (2022) [44]	Norway	Action research	Users of specialised mental health and substance abuse services	Service co-production partnerships	Specialised mental health and substance abuse unit
Selvin et al. (2021) [45]	Sweden	Interviews	Professionals working in forensic psychiatric care	Patient participation	Forensic psychiatric care

During the analysis, the condensed meaning units related to user-provider partnerships identified in the papers could be grouped in 63 distinct categories. Out of these categories seven major themes arose (see Table 3).

**Table 3:** Themes and corresponding categories that resulted form the conventional content analysis method

**Themes**	**Description of user-provider relationships**	**Implementation of user- provider partnership**	**User- provider interaction process**	**Power**	**Organisational readiness**	**Boundaries to be considered**	**Culture of care**
**Categories**	What can be considered good care	Tools and means to facilitate partnerships	Service users' willingness to participate	Class differences between user and provider	Time/work pressure limiting the ability to allow participation of users	Ability, capacity, and mental state of the user to participate in care	Importance of participation
	What is participation	Process of change	Relationship between provider and user	Power of the provider over the user	Organisational structure for change	Risks of user involvement	Knowledge base for care
	What is involvement	Bottom up approach	Collaboration between user and provider on care provision	Power of the patient	System restrictions	Protection of the provider	Mental health stigma
	Person centredness	How to integrate participation in current practices	Understanding the patient	Equality between patient and provider	Network of providers to facilitate partnerships	Consequences of user participation	Flexibility in services
	Standardisation of care	In what ways can a user be involved in care	How can a user and provider share decisions	Independent user supporter	Tensions in the organisation	Continuity of partnership in the care pathway	What leads to favourable provider attitudes for involvement
	Lack of consensus on what involvement is	Creating opportunities	Transparency of the care process	Formalisation of partnerships	The role of the manager	Flexibility in services	What leads to unfavourable provider attitudes for involvement
		How to share decision making	Creating normality		Care setting influence		Users' negative care experiences
		Training/educating to enable participation	How to facilitate the user to participate		Descriptions of the job role		Identity of the provider
		Create positive examples	Users' vs providers' expectations and needs				Attitudes shape the process
		Provision of information	Common understanding between user and provider				Barriers for the user to participate
		How to sell the participation plan	Ground principles for good user-provider relationships				What is the current treatment model
		Issues with change processes	Providers' role as responsible and facilitator				
			Communication between user and provider				
			Listening to the user				

### Description of user-provider relationships

The theme ‘descriptions of user-provider relationships’ describes what common phrases in user participation mean. It reflects on the differences in nuances between involvement, participation, person centredness and ‘good care’ discussed in the analysed papers. Overall, it was found that no clear consensus on how user-provider partnerships can be classified was available. One evolutionary concept analysis that defined the concept of service user involvement in mental healthcare was identified. In this Millar, et al. [35] propose the following definition for service user involvement:

‘An active partnership between service users and mental health professionals in decision making regarding the planning, implementation, and evaluation of mental health policy, services, education, training, and research. This partnership employs a person-centred approach, with bidirectional information flow, power sharing, and access to advocacy at a personal, service and/or societal level.’ Millar et al. [[35], p.216].

The analysis concluded that the concept of user involvement was not yet fully developed as a concept in the field. In line with this was the reported lack of consensus on what user involvement is [25, 26]. For example, in some cases user involvement was considered to be the opportunities and rights of the user to be involved in decisions about their own care [35], but in other cases user involvement was also the choice not to partake or decide on their care [27]. Similarly underlining the need for an established concept was that user participation was interchangeably used with user involvement. For providers user participation was different from traditional care models because it calls for a different approach to users, one where the experience of the user needs to be at the forefront [37].

### Mechanisms for user-provider partnerships

#### Implementation of user-provider partnerships

User participation was discussed in two ways in the selected papers, firstly it was discussed how staff perceived user participation to take place, and secondly how user participation was being used in care services. Service user participation was considered to be a valuable part of mental healthcare provision by staff [36, 40]. The largest encountered issues in the change process were related to the staff willingness to include users. In cases where implementation was reported to be superficial or just an administrative add-on [35, 41], staff where less likely to respond positively to the process around user involvement [30]. Staff took up a lot of time problematising the intervention in Ellegaard et al. [38], attempting to find the best way to work with the intervention. In the identified papers there were examples of users acting as researchers [32, 42], users’ involved in the design of an application supporting recovery [39], and of users as figureheads for treatment [36].

Integrating user participation into current care models can be done in diverse ways and encounters different challenges depending on the situation. Ellegaard et al. [38] thoroughly discussed the implementation process for patient participation for a patient-controlled admission programme. Here, it was concluded that staff juggled an interaction between managing their work duties and managing the new user-led care situation. Initially staff felt unease with the suggested changes, as the model did not fit into their current practices. This unease created a need to adapt the programme in a way that worked for their setting, seeing the potential benefit. When after some time, staff felt comfortable with the new way of working, they started to explore and shape their new way of working to their and their users’ needs [38]. Schon, et al. [30] discussed the implementation steps of a shared decision-making tool. The implementation process involved extensive training, a system of responsibility and delegation within the staff pool, followed by feedback opportunities. The implementation of the tool in Schon, et al. [30] was unsuccessful. The managers were unable to appoint local facilitators, which meant there was little ownership of the suggested implementation of the shared decision making tool. The lack of facilitators limited the opportunity for staff working with the tool to be followed and supported. This lack of follow-through meant that staff deprioritised the tool and forgot using it.

To successfully engage in user-provider partnership, both staff and user need to be involved as the success is contingent on each other’s participation [26]. On the other hand, opportunities for engagement through social contexts were created in treatment transitions, where sharing a decision can facilitate more active engagement [27]. To further create feelings of support for user participation, projects aiming to strengthen user partnerships should not merely superficially claim to be a project of user partnership, but show ownership of the partnership component [35]. This can for example be achieved through a bottom up approach showing the interest from the users or staff [30, 32, 38] and through equality of information [24, 26–28, 30, 33, 35].

A final component to consider when implementing user-provider partnerships is how to train users and providers. Bentley, et al. [37] and Brooks, et al. [41] designed and implemented training protocols so staff would be able to have users participate in their own care. The training reinforced person-centred attitudes, and an understanding of the need to facilitate involvement. In Schon, et al. [30] the training provided a willingness of staff to engage, but this tapered off when returning to normal work settings. Training and learning sessions showed the pitfalls that were later encountered in the work processes around the implemented tools.

#### User-provider interaction process

Relationships between users and providers are the basis for user-provider participation. The large majority of papers discuss relationships between users and providers [24, 26, 27, 29, 31, 34, 37, 38] and relationship building [26, 29, 33, 37, 41]. Building these relationships is essential and to do so, trust and openness are considered by both users and staff to be important conditions [27, 29, 33, 35, 42]. Users report needing to feel respected by staff, which staff should show by listening to the users [28, 29, 35, 42]. Users reported that not being listened to, was a large barrier for wanting to participate in their care [27, 33, 36, 42]. Staff on the other hand, reported needing honesty from users for these relationships to function [27, 38, 42].

This leads to another frequently discussed aspect of user-provider relationships, namely the differences in expectations and needs between users and providers. For example, in Hamann, et al. [24] patients valued the implementation of the agreed upon plan and the role of honesty and openness in the informing and feedback process, but the providers highlighted the users’ preparation for consultations and that they want to be treated politely and respectfully by users. Similar discrepancies can be found in Gunasekara, et al. [42], where users described an ideal mental health physician as empathic and emotionally involved with users, but physicians stressed the need of discerning between affective and cognitive empathy, with physicians engaging in cognitive empathy to protect themselves. However, this cognitive empathic approach was described by users as ‘distant’ and not meeting users’ needs. Other differences in expectations can be found in the duration of consultations [42] and what aspects and conditions are part of a user-provider care consultation [25, 33, 35]. There is a reported gap between users’ ideal and current practice [30, 34], followed by a desire of users that staff adapt behaviour to their needs appropriately [33, 41, 42].

As a basis for user-provider relationships, it was discussed that a mutual understanding ought to be established. Staff should take time to get to know the user and understand them [29], but also take time to create a mutual understanding of what user participation means [24, 26]. For these relationships, it was discussed that the provider ought to take the role as facilitator, as it was considered part of their job description [24, 26, 28, 37, 42]. In contrast to this, many providers discussed needing a willingness from users to engage with them. Providers expected users to be open and honest, involved, engaged, and willing to work with them and the treatment plans [24, 27, 28, 35, 37].

Collaboration on the development of treatment plans was frequently discussed. Users expressed the importance of their treatment plans and their desire to collaborate on it, however, providers did not always prioritise the importance of them [25]. Users described collaboration on goal setting, and specifically breaking these goals down into smaller steps, to be a large opportunity from the partnership to improve their recovery [29, 31, 34]. Providers stated that collaboration ideally is a natural part of the process and relationship [37], but also highlighted the importance of factors such as the users’ experience with their illness and evidence of them having employment, which helped facilitate the collaboration [24, 40]. Thus, highlighting the importance of understanding the user and acknowledging their expertise [29, 33, 41, 42].

For participation and relationship building to happen, communication was considered to be an important precondition. Communication was used to reach compromises on decisions [24]. Overall, communication was discussed in a general conceptual way, or as being ‘open’ and ‘positive’ [24, 26, 28, 29]. Few papers discussed communication within the care setting and the expectations that can be created through appropriate communication [37, 39].

#### Power

Within the care relationship the provider has authority over decision making involving the user, this leads to a power imbalance between the user and provider. This imbalance is mainly reported by users, who say that they struggle to be seen as a competent and equal person [28, 29]. Providers have power over users because they are able to decide on treatment measures that can change users’ lives [37, 40, 42]. This sense of power can be reflected in paternalistic treatment attitudes, where providers decide for the user what the best course of action is [24, 28, 30, 41]. Adding onto the power providers have in treatment decisions, is the societal class differences that widen the equality gap between users and providers [24, 41, 42].

Users have some power in the user-provider dyad and can be given more when providers create appropriate circumstances. Frequently discussed was the idea of allowing users to have responsibility and ownership over decision making about their own treatments [26, 29, 38, 39, 42]. By being actively engaged in their own care or by being provided with a care plan were ways users could regain control [33, 34]. Care plans, agendas, and contracts can be considered formal documents that users can refer back to when talking to providers. It gives users some power in the relationship [28, 31, 38, 39, 42]. However, users expressed a need for an independent figure that can support them through the care systems and that can provide them with knowledge that could create equality between user and provider [28, 33, 35]. The need for equality between both parties was called for, as it facilitated better relationships and communication concerning care [31–33, 35, 38, 42].

### Barriers and facilitators

#### Organisational readiness

The structure of an organisation is largely reflected in the culture of care provision, this means that decision-making hierarchies reflect a readiness for user participation uptake. The decision-making structures needed to enable user participation, includes formalisation of the decision-making ability of users, but also providing a clear prioritisation towards user participation within the organisation [29]. Because the staff that should be engaging with the user were not involved in decision making in the organisation themselves, user participation was considered an impossibility by them [25]. This was accurately described by a user as:

‘one of the problems is you’re working in a culture which is very top down, the people at the top make decisions and you just have to co-operate with them. Erm, I don’t think the nurses actually have much idea of what it’s like to be involved in decisions about their own lives and, therefore, don’t know how to involve the service user’. Terry and Coffey [[25], p. 962].

Situations, like described above, highlight the need to investigate the link between the organisational structure and culture in the context of user involvement [29, 34, 37]. This link was also discussed in relation to the tension it created between the old and the new practice, where the misalignment of user involvement with the traditional medical model inhibited staff to fully take up the user involvement [37, 38, 41].

The organisation also needs to have the right structures in place allowing for change processes to happen, this ranged from technical readiness [30], to addressing equality issues within the organisation [29, 35], and ensuring the right structures for service delivery are in place such as flow streams and information sharing with internal and external stakeholders [28, 30, 32, 33, 36, 38]. Furthermore, there was a reported need for top down support, ample time and a central figure that understands implementation processes to lead the change towards user involvement.

The setting where care is provided, and the associated job roles are important factors to consider in relation to user participation change processes. Within outpatient care, for example, more involvement can be possible than in acute care settings [24, 26, 37, 41]. The task load of the job roles does not always fit with the idea of user involvement and needs redefinition [25, 26]. In papers where change processes around implementing user involvement were discussed, insecurities of staff around the redefinition of roles was mentioned [38].

#### Boundaries to be considered

Providers and users agree on the influence of the users’ mental state in their ability to engage in participatory practice. Users report that having a poor mental state reduces their desire to engage in participation, in these instances, users express a need for providers to make decisions for them [24, 27]. Providers overall mention concerns with users’ mental state, expressing that their mental state might limit the users’ ability to fully comprehend the situation they are making decisions for [24, 27, 28, 30–35, 37, 41, 42]. This attitude of providers towards user’s mental state was considered in need of change by some, as users mental state is not fixed but subject to change [24]. Users’ agree with this, emphasising that they have the knowledge and skills to manage their own health and that they want to be recognised as being able, but that their ability can be limited because of temporary mental states [28, 33].

User participation comes with risks and consequences that should be considered. In Klausen, et al. [27] a balance between what the user wants and what the provider judges best was discussed. Some decisions the user makes can set back their treatment and as they lead to unpredictability of treatment. There are wider organisational implications of user participation than those for the current care setting alone [38]. These implications should not be a reason to not let users decide, but rather a consequence that needs to be adopted into care. The positive reported effects of user participation were that it empowered users [36], changed their identity [39] and gave them confidence [28, 32]. From a provider’s perspective, it was felt that providers needed to be given power from the system to decide on user participation [26, 30]. They also highlighted a need to establish boundaries with users to ensure providers private lives were not intruded upon [34, 42].

The way services are provided were adapted to fit the consequences of user participation. The partnership needs to be pulled through the whole care pathway and ensure continuity of care [27, 33, 35]. This also meant allowing flexibility in care provision and the organisation, as user led care is individualised care in need of different decisions for each user [25, 27, 28, 34, 36, 37, 41]. Flexibility was also a requisite for formalised user participation agreements. In Ellegaard, et al. [38] the patient controlled admission program and contract led to service users requesting being admitted into the hospital in the evening to sleep in a bed and wanting to be discharged in the morning. In this case, the flexibility of the contract allowed providers to say that the user could only come in through this care pathway seven days after discharge, which was better for the user’s mental health. This type of flexibility also strengthened support for the partnership as it became more person centred [27, 38].

#### Culture of care

Provider identity and attitudes shape how user participation takes place. Having an open, positive attitude towards the user facilitated a good relationship [29, 36]. In cases where the provider approached the patients and treated them as an equal or as a person, open communication and mutual respect for the circumstances was created [24, 26, 28]. A need for a change of attitude towards users was called for, one that allows users to participate in care [29, 30, 39]. Users reported negative experiences with care provision where they experienced feelings of powerlessness and coercion through forced hospitalisation [24, 29, 42], but also reported negative experiences where users were being silenced [27], or treated wrongly which led to suspicion of the provider [28]. These types of behaviours in treatment can lead to a lack of trust in the provider and suspicion, limiting willingness of users to participate in care, furthermore, these negative provider behaviours set the wrong expectations for future care [29, 36, 41, 42]. These attitudes were according to providers closely linked to their training and professional identity [37, 42].

In line with the recovery philosophy, care should be considered with the recipient as a person [31, 34]. However, systematic decision making without user involvement was reported to be the current treatment model [25, 37, 41]. Users reported that they were not being considered or talked to [25, 33], but that they have a desire to be seen as people and considered in the design of care [27, 28, 35, 36, 42]. Users were seen by some as a resource through their expertise with their illness [35, 39]. Through person-centred communication providers can draw on these experiences and knowledge [31, 38].

## Discussion

To successfully move towards person-centred care through user-provider partnerships, the themes identified in this scoping review can aid interested parties in sucessfully establishing partnerships. Especially newly identified for mental healthcare is the importance of establishing a relationship with a user that overcomes the inherent power imbalance between a physician and their user. This, mainly by users indicated, barrier limits the creation of trust that is needed for a partnership to arise.

The concept of what a user-provider partnership is, was not clearly defined in the identified literature. Only one agreed upon classification of what user-provider partnerships are could be identified. The proposed concept defined “patient involvement” as being a true partnership, which in the passivity of the word involvement seems to be a contradictory description of a true partnership.

More agreement in studies could be found as to the mechanisms needed for user-provider partnerships to arise. Most papers stated that partnerships are contigent on the participation of both the user and provider. One key finding here is the need for a respectful relationship based on trust, openess and listening to each other. This relationship can be hindered by the power imbalance between user and provider, because a provider has the ability and authority to make life altering decisions for the user. To shift the power back towards the user in this relationship, formalised contracts were proposed as a tool to manage this power imbalance. These aspects of relationship building were also identified in the previously performed systematic searches of the mental health literature by Bee, et al. [9] and Gondek et al. [10], with the exception of the role of power in the relationship, which was newly identified in the current review.

Within mental healthcare, power plays a large role in the building of therapeutic relationships, as there is a difference between the user and provider in their capacity to engage in a relationship. The provider’s perception of the user’s mental capacity and the reality displayed by users is misaligned. The best description of this observation named the mental capacity of the user as a changeable state, not a permanent one. This highlights the lack of transferability of medical models into mental healthcare and should be reflected in frameworks that aim to explain the process of user-provider partnerships in mental healthcare [46].

Change processes involving users call for personal flexibility, as these partnerships influence the professional role. Changing the traditional doctor-patient role division is needed because providers need to stop seeing a user as a patient, but rather as an active professional partner. This change perception demands personal flexibility of the provider. A proposed manner in which this perception can be changed was by seeing users as a resource to be utilised. This personal flexibility needs to be extended to dealing with each other, as in the in the context of SMIs, the mental state of the user has an impact on both parties. This also means that services need to be flexible in dealing with these changing situations.

The findings of this study show that there is gap between how providers see themselves and how their users perceive them in the care dyad. This calls for a cultural change, as it is evident that the focus towards person-centred care is increasing, but that this gap in perception prevents care-relationships evolving into partnerships. The shift towards seeing a patient as a person is one that asks for a new understanding of the professional role, as it conflicts with the traditional care models [46].

The professional is seen as the facilitator for partnerships, but the idea of a partnership with a user conflicts with the emotional distance towards the patient taught during education [37]. Similar findings can be identified in the co-production research in non-mental healthcare provision, where it is stated that professionals have a tendency to shift back to the traditional power and responsibility roles within a co-production relationship [47]. This means that future research could focus on changes in the professional role needed to allow for user-provider partnerships.

Certain aspects of the implementation process, such as respect and communication, were highlighted as barriers and/or facilitators to partnerships; a finding that is supported by a recent paper by Harrison, et al. [11], who found that it is often wrongly assumed that the co-design involving the relevant stakeholders overcomes implementation problems. They argue, however, that the co-design process can be used to identify barriers and facilitators in healthcare provision, an aspect that was found to be underutilised. The identified barriers from the current review can therefore aid healthcare provision, as our focus filled the gap found by [11] by providing barriers and facilitators.

One example of such a facilitator is a change agent, a well-established concept in implementation science for healthcare [48, 49]. This change agent has to be being willing to accept the new responsibility and power differences between user and provider, because without these the establishment of a partnership is difficult to achieve [50, 51]. From the increasing presence of the recovery movement and concepts of recovery in mental healthcare provision, it could be argued that there is an increased need from users to be an active part of their treatment and its design [52]. The recovery movement is now classed as a political movement, where instead of trying to change the system, its followers have created their own independent communities of independent care provision through for example, recovery colleges [53]. Professionals working in the field will therefore need to adapt their professional role to this increasing presence of person-centredness in care, if they want to be able to keep providing the services needed by its clientele.

### Limitations

As with all scoping reviews, the full scope of the field of research may not have been identified because of time limitations, nevertheless, the breadth of data sources can be sufficient to get a good overview of the current field. Even though titles were double screened by the researcher, a second reviewer might have had differing views that could have expanded the scope and reduced selection bias. The triangulation method and negative case identification should, however, support the credibility of the findings. Furthermore, during an initial literature search terminology relevant to the field was searched for, from this search it became evident that the field of research looking into user partnerships is fragmented and broad, therefore not all relevant terminology might have been identified.

The inductive approach of the conventional content analysis allowed the reviewers to consider all aspects of the topic with very limited preconceived ideas, but it also meant that this review was limited in testing towards previously suggested models for user participation [1, 9, 54]. Furthermore, it meant not using a standard charting form through the process, as the codes are derived from the data [21]. This meant that there is less structure in the meaning units derived from the papers across all papers, limiting the comparability of results to a larger number of papers. To increase the transferability of results, a review with a critical realistic approach evaluating existing models could be done.

## Conclusion

Based on the current findings, it is clear that more research is needed in the field of user-provider partnerships. The first steps towards co-desiging of services can be found, but the field remains scattered in terms of a unified terminology on what a partnership is. Within mental healthcare, we have found that a functioning partnership could be defined as follows:

A working relationship where user and provider communicate open and honestly with each other to achieve the mutual goals in decision making, where full support from the stakeholders in the organisation is provided. For this, a formalised partnership agreement is drawn up, where the expectations of both parties are described in detail. These involve boundary conditions of how to communicate with each other and what goals to achieve, but also how to ensure a good work-life balance for the provider and rules on how to act regarding the mental state of the user. This formal agreement should serve as the basis of equality for working in partnership to reach mutual goals in health service provision.

## Supplementary Information

**Additional file 1.**

## Data Availability

The datasets used and/or analysed during the current study are available from the corresponding author on reasonable request.
