# Supplementary material for: The processes involved in the establishment of user-provider partnerships in severe psychiatric illnesses: a scoping review
Source: BMC Psychiatry. 2022 Oct 26;22:660. doi: 10.1186/s12888-022-04303-5 (PMC9608879; doi:10.1186/s12888-022-04303-5)
Supplement: Supplementary file 1 — Additional file 1. [file 12888_2022_4303_MOESM1_ESM.docx]

# Supplementary material: search strategies

## Web of science:

((("mental health*" or psychiatr* or "depressive disorder*" or Schizophrenia or schizoaffective or bipolar or "eating disorder*" or anorexia or bulimia)) AND (((patient* or person* or client* or "service user*" or provider* or psychiatrist* or nurse* or specialist*) NEAR/3 (center* or participation* or partnership or co-production* or involvement*))) AND (Partnership* or "Collaborative Care" or "Shared decision mak*" or co-construction))

## PubMed (new version 2020):

(patient-centered care OR patient participation OR patient care management OR partnership* OR shared decision making OR co-design OR collaborative care OR patient activ* OR provider* OR nurse* OR psychiatrist* OR specialist*) AND (recovery OR recovery movement) AND (schizophrenia OR schizoaffective disorder OR bipolar OR major depressive disorder OR eating disorders OR anorexia or bulimia)

## PsycInfo:

1. (schizophrenia or schizoaffective or major depressive disorder* or eating disorder* or bipolar or anorexia or bulimia).ti,ab.

2. (partnership* or shared decision making or co-design or collaborative care).ti,ab.

3. 1 and 2
